# Impact of early vs. delayed initiation of dutasteride/tamsulosin combination therapy on the risk of acute urinary retention or BPH-related surgery in LUTS/BPH patients with moderate-to-severe symptoms at risk of disease progression

**DOI:** 10.1007/s00345-020-03517-0

**Published:** 2020-12-18

**Authors:** Salvatore D’Agate, Chandrashekhar Chavan, Michael Manyak, Juan Manuel Palacios-Moreno, Matthias Oelke, Martin C. Michel, Claus G. Roehrborn, Oscar Della Pasqua

**Affiliations:** 1 Clinical Pharmacology and Therapeutics Group, University College London, BMA House, Tavistock Square, London, WC1H 9JP UK; 2 Global Medical Urology, GlaxoSmithKline, Mumbai, India; 3 Global Medical Urology, GlaxoSmithKline, Philadelphia, USA; 4 Global Medical Urology, GlaxoSmithKline, Tres Cantos, Spain; 5 Department of Urology, St. Antonius Hospital, Gronau, Germany; 6 Department of Pharmacology, Johannes Gutenberg University, Mainz, Germany; 7 Department of Urology, Texas Southwestern Medical Center, Dallas, TX USA; 8 Clinical Pharmacology Modelling and Simulation, GlaxoSmithKline, 980 Great West Rd, London, TW8 9GS UK

**Keywords:** Lower urinary tract symptoms, Benign prostatic hyperplasia, Dutasteride, Tamsulosin, Acute urinary retention, BPH-related surgery, Clinical trial simulations, Relative risk

## Abstract

**Purpose:**

To evaluate the effect of delayed start of combination therapy (CT) with dutasteride 0.5 mg and tamsulosin 0.4 mg on the risk of acute urinary retention or benign prostatic hyperplasia (BPH)-related surgery (AUR/S) in patients with moderate-to-severe lower urinary tract symptoms (LUTS) at risk of disease progression.

**Methods:**

Using a time-to-event model based on pooled data from 10,238 patients from Phase III/IV dutasteride trials, clinical trial simulations (CTS) were performed to assess the risk of AUR/S up to 48 months in moderate-to-severe LUTS/BPH patients following immediate and delayed start of CT for those not responding to tamsulosin monotherapy. Simulation scenarios (1300 subjects/arm) were investigated, including immediate start (reference) and alternative delayed start (six scenarios 1–24 months). AUR/S incidence was described by Kaplan–Meier survival curves and analysed using log-rank test. The cumulative incidence of events as well as the relative and attributable risks were summarised stratified by treatment.

**Results:**

Survival curves for patients starting CT at month 1 and 3 did not differ from those who initiated CT immediately. By contrast, significant differences (*p* < 0.001) were observed when switch to CT occurs ≥ 6 months from the initial treatment. At month 48, AUR/S incidence was 4.6% vs 9.5%, 11.0% and 11.3% in patients receiving immediate CT vs. switchers after 6, 12 and 24 months, respectively.

**Conclusions:**

Start of CT before month 6 appears to significantly reduce the risk of AUR/S compared with delayed start by ≥ 6 months. This has implications for the treatment algorithm for men with LUTS/BPH at risk of disease progression.

**Electronic supplementary material:**

The online version of this article (10.1007/s00345-020-03517-0) contains supplementary material, which is available to authorized users.

## Introduction

Management of bothersome lower urinary tract symptoms (LUTS) constitutes the main focus of therapeutic interventions, including those patients who have confirmed diagnosis of benign prostatic hyperplasia (BPH) [1]. However, in patients at risk of disease progression, clinical deterioration is observed over time, with increasing LUTS severity (IPSS), reduction in maximum urine flow rate (*Q*_max_), episodes of acute urinary retention (AUR), or the need for BPH‐related surgery [2, 3]. Currently, LUTS/BPH management considers conservative, pharmacological and surgical treatments [1]. Specifically, on pharmacological treatment for men with moderate or severe LUTS at increased risk of disease progression, i.e. higher prostate volume, higher PSA concentration, advanced age, higher PVR, lower *Q*_max_, etc., the initial treatment of choice is a 5α-reductase inhibitor (5ARI) with or without an α-adrenoreceptor antagonist (α-blocker) or a phosphodiesterase 5 inhibitor (PDE5I) [1, 4]. In some cases, patients experiencing failure of pharmacological treatment or symptom deterioration may require minimally invasive or surgical procedures [5, 6].

While epidemiological data show that LUTS/BPH patients at risk of disease progression represent a significant proportion of the overall patient population [7], clinicians continue to use α-blocker monotherapy as a first-line treatment option to primarily manage LUTS/BPH symptoms, irrespective of the underlying rate of disease progression. Such a practice appears to contrast with robust evidence and current guidelines that support the use of combination therapy (CT) of α-blocker and 5ARI for LUTS/BPH patients with moderate or severe symptoms at risk of disease progression [1]. The pharmacological basis for the use of CT relies on the fact that in addition to the effects of α-blockers on contractile properties of prostate smooth muscle, 5ARI effectively reduces the serum and intraprostatic concentration of dihydrotestosterone, causing an involution of prostate tissue. These changes ultimately lead to a reduction in the long-term risk of AUR or BPH-related surgery (AUR/S) in patients at risk of disease progression [8].

Different arguments have been identified for the underuse of CT of α-blocker and 5ARI at the time of diagnosis, in particular the effects of 5ARIs on sexual [9, 10] and mental function [11]. Yet, there is limited awareness of the fact that the impact of CT of α-blocker and 5ARI on sexual function is primarily driven by changes in the ejaculation domain and modest impairment in the satisfaction, sexual activity and sexual desire domains, which are unlikely to be of clinical relevance [12, 13]. In addition, the use of α-blocker monotherapy as a first-line treatment underestimates long-term adverse outcomes, including higher incidence of AUR and prostate-related surgery, abnormal ejaculation and intra-operative floppy iris syndrome [14, 15].

Clinical evidence suggests that the delay in initiating a 5ARI may be associated with an increased likelihood of AUR and surgery [16–18]. However, there are no accurate estimates of the effect of such a delay due to confounding factors and differences in medical practice [19, 20]. Recently, D’Agate and colleagues have shown the long-term effects of delayed onset of dutasteride and tamsulosin CT using clinical trial simulations (CTS) [21]. Their work reveals statistically significant differences in the proportion of patients who achieved clinical response (≥ 25% IPSS reduction relative to baseline) when switching from tamsulosin monotherapy at 6 months or later (79.7% vs. 74.1%, *p* < 0.001). Overall, these results support current guidelines recommendations to start CT of α-blocker and 5ARI in men who have moderate-to-severe LUTS and are at risk of disease progression. They also reflect the disease-modifying properties of 5ARIs and reinforce the importance of slowing down or even reverse disease progression [22, 23].

Here, we apply CTS to evaluate the effect of delaying the start of treatment with dutasteride and tamsulosin CT in patients with moderate or severe LUTS/BPH at risk of disease progression. Using a cohort of patients with baseline characteristics comparable to those enrolled in previous clinical trials, the incidence and time to first episode of acute urinary retention or BPH-related surgery (AUR/S) was assessed for a range of scenarios, including immediate and delayed initiation of treatment with CT. The analysis is based on a time-to-event (TTE) model that describes the time to first AUR/S, taking into account the potential effect of baseline covariate factors [24].

## Patients and methods

### Data source

The baseline clinical and demographic data used in the CTS were obtained from six clinical trials (ARIA3001, ARIA3002, ARI40002, CombAT, CONDUCT and ARIB3003). The selection of these studies was based on the fact that protocols shared similar definitions of clinical events (i.e. AUR/S), patients had comparable medical history and study data included individual level information for LUTS/BPH patients with moderate or severe LUTS (see Tables S1, S2 and S3 for details). In addition, CombAT and CONDUCT reflect current clinical guidelines [1] for the treatment of LUTS/BPH patients.

### Clinical trial simulations

**Fig. 1:**
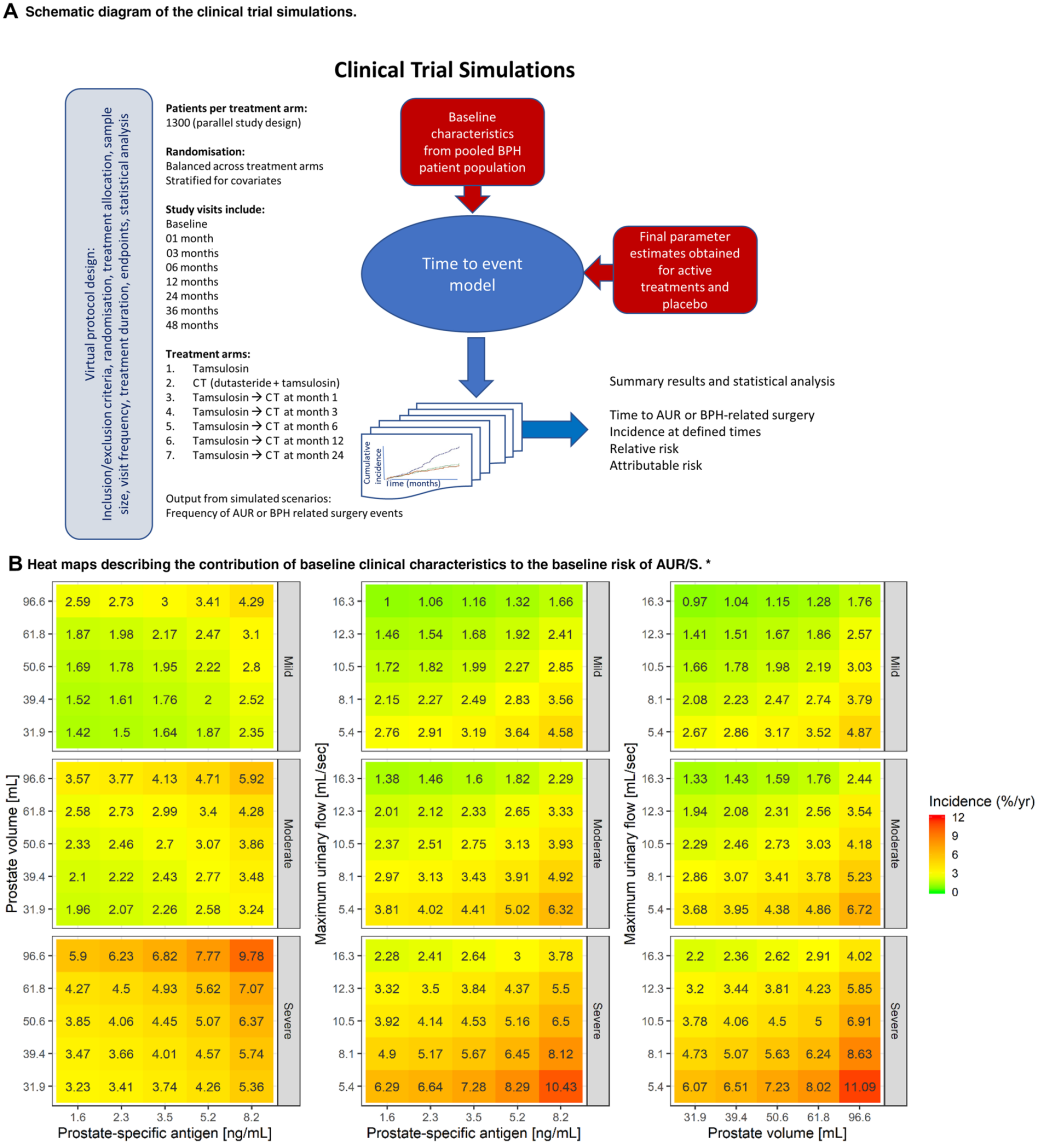

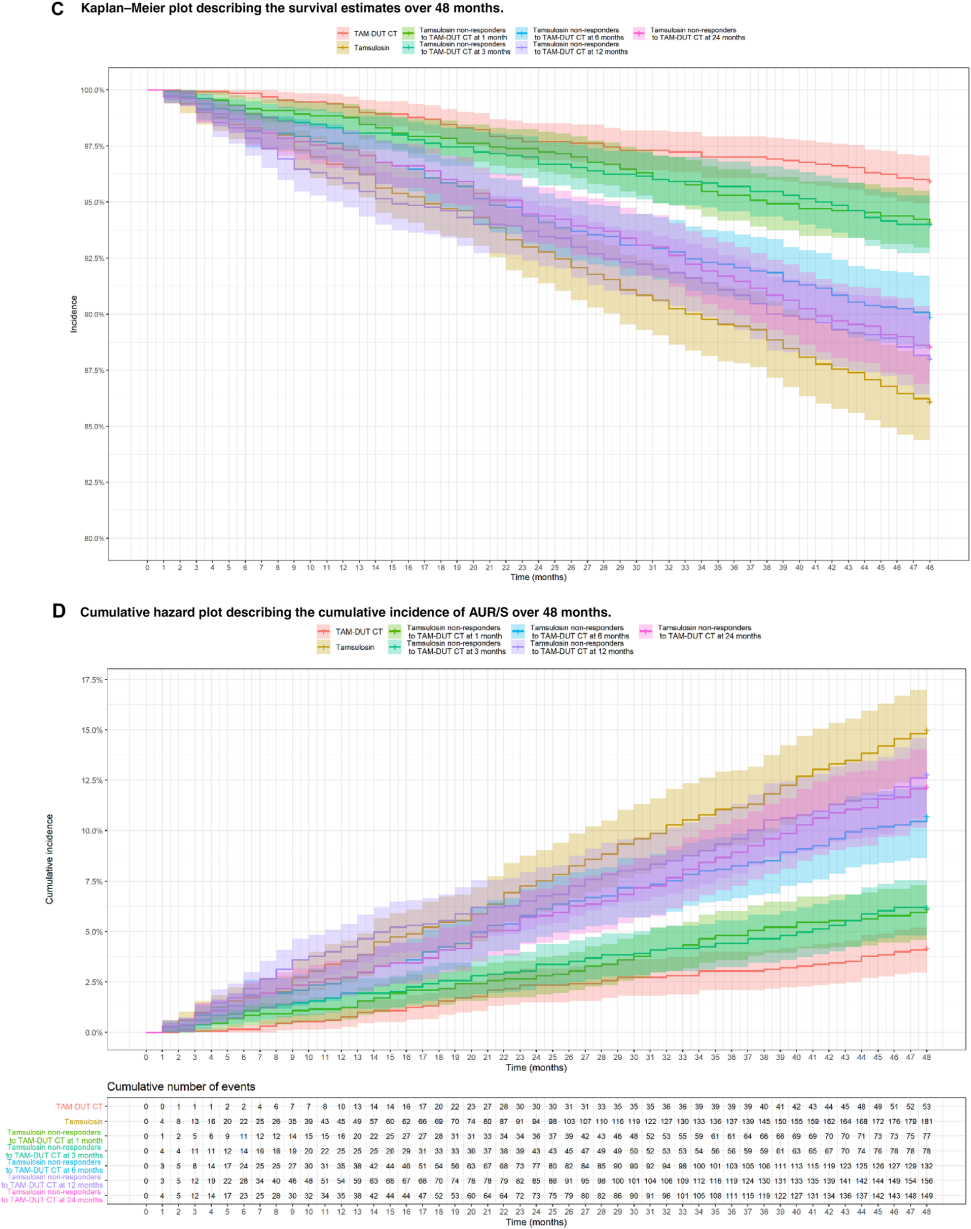
Overview of the steps for the implementation of the clinical trial simulation scenarios, covariate factors affecting baseline risk of AUR/S and main findings. **a** Schematic diagram of the clinical trial simulations based on a hazard model describing the time to first AUR/S. **b** Heat maps illustrating the contribution of baseline clinical characteristics to the baseline risk of AUR/S. * Whilst heat maps allow visualisation of the effect of the interaction between some baseline characteristics, an assessment of the baseline risk of AUR/S for individual patients, which takes into account all these factors concurrently (i.e. IPSS, PSA, PV and *Q*_max_) requires the use of the TTE model (Table S4). Even though each panel shows scales that include mild IPSS symptoms scores and normal ranges for the other baseline characteristics, defining a patient at risk of disease progression implies not only the resulting contribution of all these covariates, but also other factors than the risk of AUR/S. **c** Kaplan–Meier plot describing the survival estimates over 48 months stratified by treatment arm. Survival (*y*-axis) indicates the proportion of patients who have not had an event; at time zero the survival is 100% (i.e. no patient has experienced an AUR/S). The solid lines describe the predicted median time to first AUR/S over the period of 48 months across the different treatment arms. Shaded areas show 95% confidence intervals. The number of patients in each cohort is summarised in Table S4. **d** Cumulative hazard plot describing the cumulative incidence of AUR/S over 48 months stratified by treatment arm. Cumulative incidence of AUR/S across different treatment arms. Lines represent the median cumulative incidence of AUR/S over time. Shaded areas show the 95% confidence intervals. The table below the panel shows the cumulative number of events over time. The number of patients in each cohort is summarised in Table S4. TAM-DUT CT: tamsulosin and dutasteride combination therapy

Final parameters of the TTE model previously developed by D’Agate et al. [24] (Table S4) were used for the implementation of the simulation scenarios, which assess the potential implications of the delayed start of treatment with dutasteride and tamsulosin CT. Transition to CT was based on symptom improvement less than 25% or deterioration, as assessed by changes in IPSS relative to baseline [21]. Only non-responders to tamsulosin, i.e. patients who showed a change in IPSS < 25% from baseline after the initial treatment, were assigned to CT. From a pharmacological perspective, these scenarios represent the effect of a drug with symptomatic properties (i.e. tamsulosin) prior to the addition of a drug with disease-modifying properties (i.e. dutasteride). The CTS results were subsequently analysed using Kaplan–Meier survival curves and log-rank test. An outline of the clinical trial simulation workflow is shown in Fig. 1a. Additional details of each simulation scenario and protocol design characteristics are summarised in Table S5.

## Results

Figure 1b shows the contribution of demographic and clinical baseline characteristics to the risk of AUR/BPH-related surgery at onset of treatment. An overview of the baseline characteristics of patients included in each treatment arm is presented in Table S6.

### Effect of early vs. delayed onset of treatment with dutasteride and tamsulosin CT

The simulated scenarios reveal the effect of delayed onset of treatment with CT on the risk of AUR/S. Having taken into account the contribution of baseline characteristics, no other factor than the delayed onset of treatment with CT was identified, which explains the increased risk in patients switching from tamsulosin after 6, 12, or 24 months. The number of subject switching to CT at the different visits is summarised in Table 1, along with the number of events at the end of the study and 90% CIs from ten trial replicates. The relative and attributable risks are also shown for each treatment arm, with tamsulosin and dutasteride CT as reference arm. These results are complemented by estimates for a single trial replicate (Table S7).

**Table 1:** Proportion of patients who switch to combination therapy and summary of the results from 10 clinical trial replicates for the different treatment arms

Treatment duration	Transition to CT
Start of treatment	0
01 month	1272 (1269, 1280)
03 months	1111 (1102, 1139)
06 months	716 (700, 753)
12 months	451 (424, 472)
24 months	416 (395, 439)
36 months	0
48 months	0

Treatment arm	Number of events	Incidence at 4 years (%)	Relative risk	Attributable risk	Time to comparable progression^a^ (months)
Combination therapy (CT)	60 (51.9, 71.7)	4.6 (4, 5.5)	–	–	48
Tamsulosin	173* (153.4, 188.5)	13.3 (11.8, 14.5)	3.00 (2.25, 3.37)	8.8 (6.9, 10)	16 (12, 22.2)
Tamsulosin non-responders to CT at 1 month	67^NS^ (61.4, 81.6)	5.2 (4.7, 6.3)	1.05 (0.94, 1.57)	0.2 (−0.3, 2.3)	43 (31.5, 48)
Tamsulosin non-responders to CT at 3 months	86^NS^ (78.4, 95.8)	6.7 (6, 7.4)	1.45 (1.20, 1.72)	2.0 (1.1, 2.9)	30.5 (13, 38.1)
Tamsulosin non-responders to CT at 6 months	124* (101.1, 142.8)	9.5 (7.8, 11)	1.92 (1.61, 2.53)	4.8 (3.0, 6.5)	22.5 (14.5, 26.1)
Tamsulosin non-responders to CT at 12 months	143* (124.8, 162.0)	11.0 (9.6, 12.5)	2.32 (2.02, 3.13)	6.1 (5.1, 8.5)	17 (11, 24.6)
Tamsulosin non-responders to CT at 24 months	146* (136.7, 171.5)	11.3 (10.5, 13.2)	2.56 (2.00,2.89)	6.9 (5.4, 8.6)	16.5 (12.5, 19.5)

Upper panel: Overview of the patient population that switches to combination therapy (CT) due to non-response to tamsulosin monotherapy, as defined by a change in IPSS < 25% relative to baseline. Lower panel: number of events, incidence, relative risk, attributable risk and time to comparable progression for 10 trial replicates. Results are shown as medians (90%-confidence intervals)

^*^p < 0.001 log-rank test on survival curve; Bonferroni-corrected *α* = 0.0083

^a^Time at which the incidence of AUR/S is comparable to that observed at 48 months after immediate start of treatment with tamsulosin and dutasteride CT

Figure 1c shows the Kaplan–Meier survival curves in each treatment scenario. The different curves indicate that switching treatment from tamsulosin to CT at 6 months or later from the start of treatment has a significant effect on the proportion of events at completion of the study at month 48. As summarised in Fig. 1d, the impact of the delayed onset of treatment with CT is also reflected in the cumulative hazard plot. Along with it, the figure shows the cumulative number of events for each treatment scenario.

## Discussion

Currently, LUTS/BPH management considers conservative, pharmacological and surgical treatments. The strength of each recommendation is determined by the balance between desirable and undesirable consequences of alternative management strategies and the quality of the evidence. Whilst guidelines are available, the choice of treatment should be reached in a shared decision-making process between the physician and patient [1, 25, 26].

Specifically, for men with moderate or severe LUTS at risk of disease progression, 5ARI with an α-blocker is an initial treatment of choice recommended by clinical guidelines [1]. Nevertheless, in clinical practice patients with moderate or severe LUTS at risk of disease progression continue to be treated initially with only an α-blocker [27]. Little attention has been given to the impact of variable disease progression rates on LUTS deterioration and subsequent implications for the risk of AUR/S following delayed initiation of 5ARIs [28, 29].

Even though the risk and incidence of complications may vary due to the contribution of different risk factors, AUR, which often presents as an emergency, remains an important complication for patients at risk of disease progression with financial, emotional and health-related consequences [30, 31] On the other hand, BPH-related surgery is primarily a consequence of the perceived severity of the condition. In fact, irrespective of considerable variation between studies in the reported incidence of AUR in male patients, AUR results in prostatectomy in only 24–42% of men [28], while those who avoid surgery through a successful trial without catheter were found to be at high risk of requiring surgery within a year [29].

The benefits of CT of α-blocker and 5ARI for this group of patients have been evaluated extensively in different investigations, which have also shown a statistically significant reduction in the incidence of AUR/S [32, 33]. These findings are further supported by a large retrospective study, which identified that patients who received dutasteride following a urologist referral had a lower risk of BPH-related prostate surgery than those treated with finasteride [30]. In line with the aforementioned findings, the results from our simulations indicate that tamsulosin does not reduce the risk of AUR/S, and that delaying the start of treatment with CT by ≥ 6 months results in a statistically significant increase in the incidence of events.

Using scenarios which reflect a real clinical trial setting where patients are often randomized to different treatment arms, it was possible to demonstrate that drugs with disease modifying properties reduce the risk and incidence of AUR/S. Early onset of treatment with dutasteride and tamsulosin CT (i.e. < 6 months delay) leads to approximately three-fold decrease in relative risk compared to tamsulosin [22]. This effect wanes progressively with delayed transition from tamsulosin to CT; the longer the delay, the higher the incidence of events. This benefit is complemented by the effect of dutasteride and tamsulosin CT on symptom deterioration as assessed by IPSS. Early onset of treatment with tamsulosin and dutasteride CT does not only result in a significantly higher responder rate relative to tamsulosin (*p* < 0.001); it also shows a larger proportion of patients with larger LUTS improvement (i.e. ≥ 50% change in IPSS relative to baseline) than when CT is delayed by ≥ 6–24 months (60.8% vs. 48.4–52.7%) [21].

Our analysis also shows that baseline characteristics affect baseline hazard rate and as such contribute to the instantaneous risk (Fig. 1b), but are not predictive of the overall response to an intervention, which is determined by treatment type. In fact, baseline demographic and clinical characteristics in non-responders to tamsulosin do not differ significantly from patients on CT. This implies that the risk of progression at the time of diagnosis will be miscalculated if only baseline characteristics are used to predict treatment response.

From a methodological perspective, we acknowledge that to address the key research question from this investigation, it is essential to discriminate the contribution of multiple interacting factors to the instantaneous risk of AUR/S, including baseline covariates, trial design and treatment type. Whereas these factors may not be easily controlled in a prospective clinical trial, CTS do offer an opportunity to control and eventually assess the effect of confounding or uncontrolled factors [34]. In this regard, it should be emphasised that it may not be possible to accurately assess the magnitude of the effect of delayed start of CT on the incidence of AUR/S based on a prospective or retrospective clinical trial. In addition to the large sample size and logistic challenges associated with patient monitoring and follow-up, prospective clinical studies may not be considered ethically acceptable, especially when guidelines recommend it for men who have moderate-to-severe LUTS and are at risk of disease progression (i.e. higher prostate volume, higher PSA concentration, advanced age, higher PVR, lower *Q*_max_, etc.) [1]. Likewise, any attempt to use retrospective data from randomised controlled clinical trials or real-life clinical settings will be fraught with difficulties, as one needs to consider the effect of censoring and other deviations, which cannot be easily accounted for during data analysis. These limitations are illustrated by a recent investigation on the effects of early (≤ 6 months after starting any medical treatment for BPH [baseline]), intermediate (between > 6–24 months from baseline) and late (24 months after baseline) initiation of add-on dutasteride therapy on the incidence of AUR/S in Japanese patients with moderate-to-severe BPH [18]. The relatively small sample size and striking differences in the incidence of BPH-related surgery across sites have resulted in confounding and consequently made it very difficult to disentangle the effect of varying medical practice from delayed start of CT on overall treatment outcome.

Hence, the differences observed across CTS scenarios may have further relevance in real life. Considering the chronic nature of the disease, on a longer time scale the effect of disease-modifying properties of dutasteride cannot be compensated by symptomatic interventions. Patients who are eligible to initiate CT miss the benefit over long term, as shown by the difference in the cumulative incidence, relative and attributable risk in treatment arms with patients who switch from tamsulosin at ≥ 6 months.

### Limitations

Undoubtedly, there are limitations in our work. Whereas the protocol conditions and criteria outlined for the evaluation of early and delayed onset of treatment with CT may not be easily implemented in real life due to ethical and practical challenges, assumptions had to be made regarding trial characteristics, model parameter precision, and generalisability of the findings from the different simulation scenarios. An overview of the main assumptions and limitations is summarised in Table S4. Moreover, it should be noted that prior to implementing the simulation scenarios, an attempt was made to assess the predictive performance of the model by simulating the survival estimate over time for a subset of patients (*n* = 1405) who switched from placebo treatment (randomised phase) to dutasteride monotherapy (Fig. S1). These data were not used during model development. There were no other controlled studies in which patients on monotherapy were switched to CT.

From a statistical perspective, we have assumed no carry over effect for treatment with drugs showing symptomatic improvement only (i.e. tamsulosin). In addition, as transition from tamsulosin to CT was implemented by design, i.e. switching at pre-specified times for each treatment arm, no additional statistical methods were used for adjustment or correction of potential bias in estimates [35].

## Conclusions

The use of CTS enabled the evaluation of the implications of delayed start of CT with tamsulosin and dutasteride. Delaying the start of treatment with CT by ≥ 6 months significantly increases the risk of AUR/S relative to those who start immediately on CT. Together with previous findings from a longitudinal model describing individual IPSS trajectories, these results show that early start of CT does not only ensure higher response rate and overall symptoms improvement [36]; it also slows down disease progression, reducing the risk of AUR/S. However, such benefits need to be weighed for individual patients taking into account the risk of progression and susceptibility to the adverse events of treatment as well as patient preferences.

## Electronic supplementary material

Below is the link to the electronic supplementary material.

Supplementary file1 (PDF 280 KB)
