# Supplementary material for: Impact of early vs. delayed initiation of dutasteride/tamsulosin combination therapy on the risk of acute urinary retention or BPH-related surgery in LUTS/BPH patients with moderate-to-severe symptoms at risk of disease progression
Source: World J Urol. 2020 Dec 18;39(7):2635–43. doi: 10.1007/s00345-020-03517-0 (PMC8332595; doi:10.1007/s00345-020-03517-0)
Supplement: Supplementary file 1 — Supplementary file1 (PDF 280 KB) [file 345_2020_3517_MOESM1_ESM.pdf]

Supplementary Materials

Table S1 Overview of the studies identified for the development and evaluation of the time-to-event model used as basis for the clinical trial simulations. Protocol title is shown along with details regarding treatment type and duration and the purpose of the study data during model building and validation procedures.

Study No.	Informed consent for data reuse	Dose and Administration	Number of Patients	Purpose in the study	Visits (months or weeks)
ARIA3001	Yes	q.d. 0.5 mg dutasteride for two years	720	Model building	AUA-SI/IPSS: 0, 1, 3, 6, 12, 18, 24, 30, 36, 42 and 48<br>Prostate volume: 0, 1, 6, 12, 24 and 48<br>Max. urinary flow: 0, 1, 3, 6, 12, 18, 24, 30, 36, 42 and 48<br>PSA: 0, 1, 3, 6, 12, 18, 24, 36 and 48<br><br>AUR and BPH-related surgery* monitored throughout study and censored at four years
		q.d. placebo for two years	720	Model building	
ARIA3002	Yes	q.d. 0.5 mg dutasteride for two years	677	Model building	AUA-SI/IPSS: 0, 1, 3, 6, 12, 18, 24, 30, 36, 42 and 48<br>Prostate volume: 0, 3, 6, 12, 24, 36 and 48<br>Max. urinary flow: 0, 1, 3, 6, 12, 18, 24, 30, 36, 42 and 48<br>PSA: 0, 1, 3, 6, 12, 18, 24, 36 and 48<br><br>AUR and BPH-related surgery* monitored throughout study and censored at four years
		q.d. placebo for two years	685	Model building	
ARI40002	Yes	q.d. 0.5 mg dutasteride & 0.4 mg tamsulosin for 36 weeks	164	Model building	AUA-SI/IPSS: 0, 4, 12, 24, 30, 36 and 37 weeks<br>PSA: 0 and 36 weeks<br><br>AUR and BPH-related surgery* monitored throughout study and censored at 36 weeks
		q.d. 0.5 mg dutasteride for 12 weeks after 24 weeks. q.d. combination therapy	163	Model building	
CombAT	Yes	q.d. 0.4 mg tamsulosin for four years	1611	Model building	AUA-SI/IPSS: 0, 3, 6, 9, 12, 15, 18, 21, 24, 27, 30, 33, 36, 39, 42, 45 and 48<br>Prostate volume: 0, 12, 24, 36 and 48<br>Max. urinary flow: 0, 6, 12, 18, 24, 30, 36, 42 and 48<br>PSA: 0, 12, 24, 36 and 48
		q.d. 0.5 mg dutasteride for four years	1623	Model building	
		q.d. 0.5 mg dutasteride & 0.4 mg tamsulosin therapy for four years	1610	Model building	

					AUR and BPH-related surgery* monitored throughout study and censored at four years
CONDUCT	Yes	watchful waiting with protocol-defined initiation of q.d.0.4 mg tamsulosin	373	Model building	AUA-SI/IPSS: 0, 1, 3, 6, 9, 12, 15, 18, 21 and 24<br>PSA: 0, 6, 12 and 24<br><br>AUR and BPH-related surgery* monitored throughout study and censored at two years
		q.d. 0.5 mg dutasteride & 0.4 mg tamsulosin therapy for two years	369	Model validation	
ARIB3003	Yes	q.d. 0.5 mg dutasteride for two years	770	Model validation	AUA-SI/IPSS: 0, 1, 3, 6, 12, 18, 24, 30, 36, 42 and 48<br>Prostate volume: 0, 6, 12, 18, 24 and 48<br>Max, urinary flow: 0, 1, 3, 6, 12, 18 24, 30, 36, 42 and 48<br>PSA: 0, 1, 3, 6, 12, 18, 24, 36 and 48<br><br>AUR and BPH-related surgery* monitored throughout study and censored at four years
		q.d. placebo for two years	753	Model validation	

* An overview of the definition of BPH-related surgery is summarised in Table S2.

Table S2 Interventions encompassed by the definition of BPH-related surgery per study protocol

Intervention	ARIA3001	ARIA3002	ARIB3003	CONDUCT	CombAT*
Adenomectomy, electroresection, transurethral resection of the prostate (TURP), visual laser ablation of the prostate (VLAP), prostatectomy laser resection	X	X	X	X	X
Hyperthermia, thermotherapy, microwave, radio-frequency, transurethral microwave thermotherapy (TUMT)	X	X	X	X	X
Prostatectomy, open, partial, retropubic, transvesical, suprapubic	X	X	X		X
Balloon dilatation, prostate	X	X	X	X	X
Prostatotomy, TUIP	X	X	X		X
Prostatectomy: retropubic with or without nerve sparing, perineal, salvage, laparoscopic, laparoscopic assisted					X
Radioactive seeding of the prostate (brachytherapy), transrectal high intensity focussed ultrasound (HIFU)					X
Transurethral drainage of prostatic abscess, drainage of prostatic cysts, excision of periurethral stricture					X
Prostatic urethral stenting				X	X
Ethanol injections into the prostate					X

[¥]CombAT study protocol: BPH-related prostatic surgical and minimally-invasive interventions included but not limited to the procedures detailed above. Many minimally-invasive techniques to treat prostatic obstruction found in the CombAT trial were not available during earlier clinical trials.

Table S3 **Demographics and baseline characteristics of the pooled patient population included in the meta-analysis.**

Baseline demographics	N	Mean	SD		Median	Min	Max
Age (y)[1]	10236	66.2	7.20		66	47	94
Body weight (kg)[1]	10206	83.2	13.60		82	37	179
Height (cm)	10204	174.1	7.48		175	132	208
Baseline PSA (ng/mL)	10206	3.98	2.10		3.4	0.6	23.2
Baseline prostate (mL)	9875	54.53	23.00		48.5	16.59	296.89
Baseline IPSS	10228	16.48	6.10		16	1	35
Baseline maximum urinary flow (mL/s)	9163	10.49	3.59		10.20	2.2	36.2
BMI (kg/m^2)	10210	27.44	3.99		26.91	12.36	59.73
Duration of BPH symptoms (y)	9881	5.17	4.77		4.00	0	54.8
Time from BPH diagnosis (y)	10080	2.65	4.26		2.3	0.58	52
Alcohol use (Y/N)		6198/3992					
Sexually active (Y/N)		7244/2984					
Race (White/Black/Hispanic/Asian)		9268/229/276/374					
Smoking status (Y/N)		1239/8998					
**Treatment information**	**Placebo**	**WW**	**Tamsulosin**		**Dutasteride**	**Combination therapy**	
Number of subjects	2158	373	1611		3790	2143	
Treatment duration:							
≤12 months	475	44	180		659	610	
≤18 months	638	60	296		872	694	
≤24 months	2158	373	381		1181	940	
≤36 months	2158	373	536		1625	1125	
≤48 months	2158	373	1611		3790	2143	

Table S4 Final parameter estimates for TTE model

Parameter	Parameter Estimate	RSE (%)	95%-CI
**Baseline hazard rate λ (per 100000 subjects) [day^{-1}]**	7.78	(6%)	6.86 - 8.70
**Hazard ratios**			
Tamsulosin	1	-	-
Dutasteride	0.432	(7%)	0.352 - 0.512
Combination	0.336	(7%)	0.249 - 0.423
Baseline IPSS	1.04	(19%)	1.022 - 1.050
Baseline PSA	1.08	(24%)	1.041 - 1.120
Baseline Prostate Volume	1.01	(14%)	1.007 - 1.012
Baseline Maximum Urinary Flow	0.91	(14%)	0.889 - 0.935

RSE: relative standard error; 95%-CI: 95%-confidence intervals

Table S5 **Protocol design characteristics used for the simulation* of AUR/S and individual IPSS trajectories after immediate and delayed start of treatment with tamsulosin-dutasteride combination therapy:**

**Protocol characteristics**	
**Endpoints:**	Endpoints evaluated were: 1) time to AUR or BPH-related surgical intervention; 2) incidence of AUR or BPH related surgical intervention (at different time points relative to the start of treatment); 3) increase in the time to AUR or BPH-related surgery due to combination therapy; 4) relative risk of AUR or BPH-related surgery in patients immediately treated with combination therapy, as compared to alternative treatments, namely tamsulosin monotherapy and delayed onset of combination therapy; 5) attributable risk due to the delayed onset of treatment with tamsulosin and dutasteride combination therapy (CT).
**Simulation scenarios**	Simulation scenarios were used to evaluate the long-term effect of different intervention on time to AUR/S in BPH patients with moderate or severe symptoms at baseline. To ensure simulations reflected the original clinical trial population, baseline characteristics from the available pooled population (**Table S2**) were resampled and used as input in each of the simulation scenarios. In addition, re-sampling of patients into each clinical trial scenario was performed taking into account the observed covariate distributions observed in the available clinical studies. Given the large sample size used across the different scenarios, along with the prior evidence of the predictive performance of the model, results from 10 trial replicates were assumed to be sufficiently accurate and precise to assess eventual differences between scenarios. Confidence intervals from trial replicates based on resampling from the same patient pool were deemed appropriate to account for model parameter uncertainty.<br><br>In an attempt to mimic clinical practice, a parallel study design was used, i.e., each patient was randomised to one of the treatment arms at the beginning of the trial. The transition from tamsulosin to CT in predefined study arms was based on LUTS symptom deterioration. Only non-responders to tamsulosin were assigned to CT, as assessed by the relative changes in IPSS scores at the predefined visit. Treatment response (LUTS improvement) was defined as a drop in IPSS score of ≥25% from baseline. Consequently, a non-responder is a subject who shows a change in IPSS score of < 25% relative to baseline. Except for baseline values, which were resampled from the original patient pool, individual IPSS values were derived by simulation using the drug-disease model developed previously [1]. In addition to the incidence of events, Kaplan-Meier curves, relative risk and the attributable risk of AUR and BPH-related surgery were also evaluated.
**Baseline characteristics:**	The patient population randomised into each simulation scenario was based on the inclusion and exclusion criteria used in the CombAT and CONDUCT studies [2,3]. Inclusion criteria comprised the following requirements at baseline: men ≥50 years of age, IPSS ≥8 points, prostate volume ≥30 mL, total serum PSA ≥1.5 ng/mL and <10 ng/mL, Q_{max} >5 ml/s and ≤15 mL/s and a minimum voided volume ≥125 mL. Exclusion criteria included history or evidence of prostate cancer, previous prostatic surgery, history of AUR within 3 months prior to study entry, 5ARI use within 6 months (or dutasteride within 12 months) prior to entry or use of an α-blocker or phytotherapy for BPH within 2 weeks prior to entry.
**Sample size estimation**	Sample size calculations were based on the estimates of AUR/S incidence at month 48, as reported by Roehrborn and colleagues [3]. Post-hoc sample size was computed under the assumption of an incidence of 4.2% for CT. A conservative sample size of

	1300 subjects per arm (total: 9100 subjects) was chosen to detect a statistically significant and clinically relevant difference of 3% in TTE rate between treatment arms at month 48. Considering the aforementioned effect size, 0.05 level of significance and 90% statistical power, the minimum sample size required for the analysis was 1260 subjects per arm.
**Study visits**	Simulation scenarios were based on typical clinical visits and included follow-up over a period of 48 months. This period was used to avoid empirical extrapolation beyond the observation window considered for model development and validation, for which there is also supporting clinical trial data. Visits including IPSS measurements were at study entry (baseline) and at 1, 2, 3, 4, 5, 6, 12, 18, 24, 30, 36, 42 and 48 months after the start of treatment. Screening measurements were not included or considered for simulation purposes. To minimise censoring and reduce eventual bias when tabulating the results, additional sampling times were simulated at bi-weekly time intervals for the evaluation of occurrence of AUR/S events.
**Treatment arms**	For this analysis, patients were assumed to have undergone a washout period prior to the start of the intervention. In addition, the assigned treatments (0.5 mg dutasteride and 0.4 mg tamsulosin) were initiated as CT or tamsulosin monotherapy under the assumption of varying intervals or delay after BPH diagnosis, as in the original patient population. One reference and six alternative treatment scenarios were evaluated, namely:<br><br>**Scenario 1** (reference): CT over 48 months;<br>**Scenario 2:** tamsulosin over 48 months;<br>**Scenario 3:** tamsulosin until visit 2 (1-month delay) followed by CT up to month 48;<br>**Scenario 4:** tamsulosin until visit 4 (3-month delay) followed by CT up to month 48;<br>**Scenario 5:** tamsulosin until visit 7 (6-month delay) followed by CT up to month 48;<br>**Scenario 6:** tamsulosin until visit 8 (12-month delay) followed by CT up to month 48;<br>**Scenario 7:** tamsulosin until visit 10 (24-month delay) followed by CT up to month 48.<br><br>Only non-responders to tamsulosin switched to CT at the selected visits.
**Statistical methods:**	Simulated events (AUR/S) were described by Kaplan-Meier survival curves and analysed using a log-rank test. Survival curves are a standard way to represent the occurrence of an event over a predefined observation window [4]. In the context of this analysis, survival refers to the proportion of patients who have not had an AUR/S. Median estimates and 90% confidence intervals are presented in tabular format for results from trial replicates (n=10). On the other hand, to ensure direct comparison with data arising from a prospective study protocol, graphical summaries are described as those obtained in a single trial, i.e., only point estimates are shown without confidence intervals.<br><br>The primary objective of the statistical analysis was to compare the difference in the incidence of AUR/S at month 48 between patients in the reference treatment arm (i.e. those who initiate treatment immediately with tamsulosin and dutasteride CT) and those whose treatment with tamsulosin and dutasteride CT is delayed. Assessment of the superiority of immediate onset of treatment with tamsulosin and dutasteride CT was based on a two-sided test with significance level α=0.05. As transition from tamsulosin to CT was implemented by design, i.e., with transition defined at pre-specified times for each treatment arm, no additional statistical methods for adjustment of estimated treatment effect were used to correct for potential bias in estimates due to patients switching from their allocated treatment [5, 6]. In addition to the incidence of AUR/S at month 48, attributable risk, relative risk and time to reach

	comparable incidences at 48 months following different interventions were also calculated.<br><br>Given that the endpoints of the clinical trial simulations were only considered at the end of study, the hazard ratio was not estimated when summarising the results. Moreover, since the rate of events is low and the incidence of events corresponding to a survival rate of 50% of the subjects is never reached during the time frame considered for the simulations, the median time to event could not be calculated with enough precision and was therefore excluded from the analysis. Instead, the time at which the incidence of AUR/S after delayed initiation of CT reaches that of the reference group at 48 months was estimated to assess the implications of treatment with disease-modifying properties on the progression of BPH.
**Assumptions: and limitations**	• Parameter estimates obtained from the pooled patient database (N=10,234 had dosing records) were assumed to be sufficiently precise to replicate the performance of the treatment in a wider population, as observed in clinical practice. However, we recognise that inclusion/exclusion criteria may not fully reflect the LUTS/BPH population eligible for treatment with α-blockers and 5-ARI in clinical practice.<br>• Interindividual variability in pharmacokinetics was assumed to have a minor impact on treatment response, as the currently approved dose levels of tamsulosin-dutasteride yield nearly maximum pharmacological effect.<br>• All CTS scenarios were implemented under the assumption of perfect adherence to treatment. In real-life conditions, different adherence patterns may occur, depending on symptom severity and/or comorbidities, which may significantly alter the predicted differences across CTS scenarios<br>• As drop-out in real clinical trials appears to be non-informative (i.e. at random), treatment scenarios were implemented without dropout.<br>• The cumulative incidence was not calculated beyond 48 months to ensure that simulation results could be supported by existing clinical data, i.e., the time span used in the analysis matches the duration of the longest clinical trial included in the development of the model.<br>• The use of 10 trial replicates may seem limited, but given the sample size in each treatment arm (n=1300), this was deemed adequate to obtain reliable estimates of the 90%-confidence intervals of the survival function.<br>• The assessment of treatment response at each visit using predicted IPSS for each patient was based on the longitudinal model describing individual IPSS trajectories. Whilst this does not represent a limitation, the predicted response does not include residual random variability, which may be relatively large in real life.

5g-ARI, 5α-reductase inhibitor; AUR, acute urinary retention; CTS, clinical trial simulations; IPSS, International Prostate Symptom Score; LUTS/BPH, lower urinary tract symptoms due to benign prostatic hyperplasia; PSA, prostate-specific antigen; Q_{max}, maximum urine flow rate.

* Clinical trial simulations were implemented in NONMEM version 7.2 (Icon Development Solutions, MD, USA) based on the time to event model and the final parameter estimates shown in Table S3. AUR/S events were simulated for each scenario along with IPSS response over a period of 48 months. All required data manipulation, including graphical and statistical summaries were performed in R (version 3.1.1).

Table S6 **Demographics and baseline characteristics of the patient population used for the evaluation of the effect of early vs. delayed onset of treatment with combination therapy using clinical trial simulations.**

Total number of patients at each visit includes those who switch to CT. Adherence to treatment and was considered constant throughout the duration of the study. Dropout was considered zero for the purposes of the analysis.

	**Combination therapy (CT)**		**Tamsulosin**		**Tamsulosin non-responders to CT at 1 month**		**Tamsulosin non-responders to CT at 3 months**		**Tamsulosin non-responders to CT at 6 months**		**Tamsulosin non-responders to CT at 12 months**		**Tamsulosin non-responders to CT at 24 months**	
	**N**	**Mean (min - max)**	**N**	**Mean (min - max)**	**N**	**Mean (min - max)**	**N**	**Mean (min - max)**	**N**	**Mean (min - max)**	**N**	**Mean (min - max)**	**N**	**Mean (min - max)**
Age (y)	**1300**	**66.55 (50 - 91)**	**1300**	**66.47 (50 - 94)**	**1300**	**65.9 (50 - 88)**	**1300**	**66.31 (50 - 88)**	**1300**	**66.07 (48.25 - 90)**	**1300**	**66.22 (50 - 85)**	**1300**	**66.25 (47 - 89)**
Body Weight (kg)	**1292**	**84.04 (46 - 168)**	**1298**	**83.04 (45 - 171.8)**	**1296**	**83.22 (48 - 174)**	**1295**	**83.18 (51 - 179)**	**1297**	**82.51 (37 - 138)**	**1297**	**83.35 (43.5 - 148)**	**1297**	**83.07 (45.5 - 178)**
Duration of BPH symptoms (y)	**1253**	**5.15 (0.02 - 34.17)**	**1257**	**5.12 (0 - 54.83)**	**1254**	**5.25 (0.06 - 40)**	**1250**	**5.38 (0.08 - 38.92)**	**1246**	**5.54 (0.02 - 43.92)**	**1258**	**4.99 (0.02 - 30.42)**	**1258**	**4.98 (0 – 35)**
Time from BPH diagnosis (y)	**1284**	**3.55 (0 - 37)**	**1283**	**3.72 (0 - 40)**	**1282**	**3.61 (0 - 52)**	**1281**	**3.75 (0 - 40)**	**1275**	**3.79 (0 - 43.92)**	**1277**	**3.63 (-0.58 - 37)**	**1276**	**3.6 (0 - 40)**
Baseline PSA (ng/mL)	**1298**	**3.98 (0.6 - 12.8)**	**1296**	**3.92 (0.7 - 10.2)**	**1293**	**3.97 (1.5 - 13)**	**1298**	**4.06 (0.7 - 11.6)**	**1294**	**4.07 (1.3 - 10.5)**	**1297**	**3.85 (1 - 10.6)**	**1296**	**4.05 (1 - 23.2)**
Baseline IPSS	**1300**	**16.98 (8 - 35)**	**1300**	**16.88 (8 - 35)**	**1300**	**17.27 (8 - 34)**	**1300**	**17.38 (8 - 35)**	**1300**	**17.05 (8 - 35)**	**1300**	**17.15 (8 - 35)**	**1300**	**16.86 (8 - 35)**
BMI (kg/m^2)	**1290**	**27.69 (17.42 - 59.52)**	**1297**	**27.42 (15.21 - 51.36)**	**1296**	**27.44 (17.78 - 57.47)**	**1295**	**27.43 (17.65 - 56.49)**	**1296**	**27.16 (12.36 - 59.73)**	**1295**	**27.40 (16.89 - 47.47)**	**1297**	**27.42 (15.67 - 58.12)**

Baseline Prostate volume (mL)	**1300**	**54.64 (18.75 - 286.44)**	**1300**	**53.96 (19.83 - 178.37)**	**1300**	**54.62 (24.53 - 191.62)**	**1300**	**54.36 (19.97 - 180.27)**	**1300**	**54.87 (16.59 - 274.16)**	**1300**	**52.93 (24.61 - 186.68)**	**1300**	**54.29 (21.61 - 205.19)**
Baseline Maximum Urinary Flow (mL/s)	**1300**	**10.18 (2.9 - 36.2)**	**1300**	**10.02 (2.9 - 31.8)**	**1300**	**10.09 (2.2 - 31)**	**1300**	**10.04 (2.5 - 28.7)**	**1300**	**10.02 (2.6 - 29.9)**	**1300**	**9.88 (2.6 - 25.8)**	**1300**	**10.05 (2.8 - 33)**
Height (cm)	**1291**	**174.09 (142 - 202)**	**1297**	**173.92 (140 - 200)**	**1296**	**174.05 (140 - 203.2)**	**1296**	**174.05 (146 - 205)**	**1297**	**174.24 (152 - 203)**	**1295**	**174.32 (138.6 - 208)**	**1297**	**173.97 (132 - 204.5)**
Alcohol use (N/Y)	**469/822**		**513/782**		**488/806**		**522/771**		**511/786**		**518/776**		**495/798**	
Sexually active (N/Y)	**393/905**		**356/941**		**361/928**		**376/922**		**371/928**		**393/904**		**410/890**	
Race (White/Black /Hispanic/Asian)	**1193/23/37/39**		**1183/33/36/40**		**1173/23/37/49**		**1195/34/22/42**		**1177/26/40/46**		**1158/31/41/59**		**1178/31/32/47**	
Smoking status (N/Y)	**1138/162**		**1153/147**		**1132/168**		**1133/167**		**1148/152**		**1154/146**		**1147/153**	

Table S7 **Number of events, relative risk and attributable risk for different treatment arms.**

Upper panel: Overview of the patient population that switches to combination therapy (CT) due to non-response to tamsulosin monotherapy, as defined by a change in IPSS < 25% relative to baseline. Lower panel: number of events, incidence, relative risk, attributable risk and time to comparable progression for single trial replicate.

**Treatment duration**	**Transition to CT**
Start of treatment	0
01 month	1280
03 months	1104
06 months	720
12 months	448
24 months	416
36 months	0
48 months	0

Treatment arm	***Number of events***	***Incidence at 4 years (%)***	***Relative risk***	***Attributable risk***	***Time to comparable progression ** (months)***
Tamsulosin and dutasteride combination (CT)	53	4.1	-	-	48
Tamsulosin	181*	13.9#	3.42	9.8	13
Tamsulosin non-responders to CT at 1 month	77^{NS}	5.9	1.45	1.8	31
Tamsulosin non-responders to CT at 3 months	78^{NS}	6.0	1.47	1.9	32
Tamsulosin non-responders to CT at 6 months	132*	10.1#	2.49	6.0	17
Tamsulosin non-responders to CT at 12 months	156*	12.0#	2.94	7.9	11
Tamsulosin non-responders to CT at 24 months	149*	11.5#	2.81	7.4	19

*p<0.001 log-rank test on survival curve; Bonferroni-corrected α=0.0083

#p<0.001 pairwise chi-square test on incidence rate at 4 years; Bonferroni-corrected α=0.0083

** Time at which the incidence of AUR/S is comparable to that observed at 48 months after immediate start of treatment with tamsulosin and dutasteride CT.

Figure S1 **Visual-predictive check showing Kaplan-Meier survival estimate over for a subset of patients switching from placebo to open label dutasteride.**

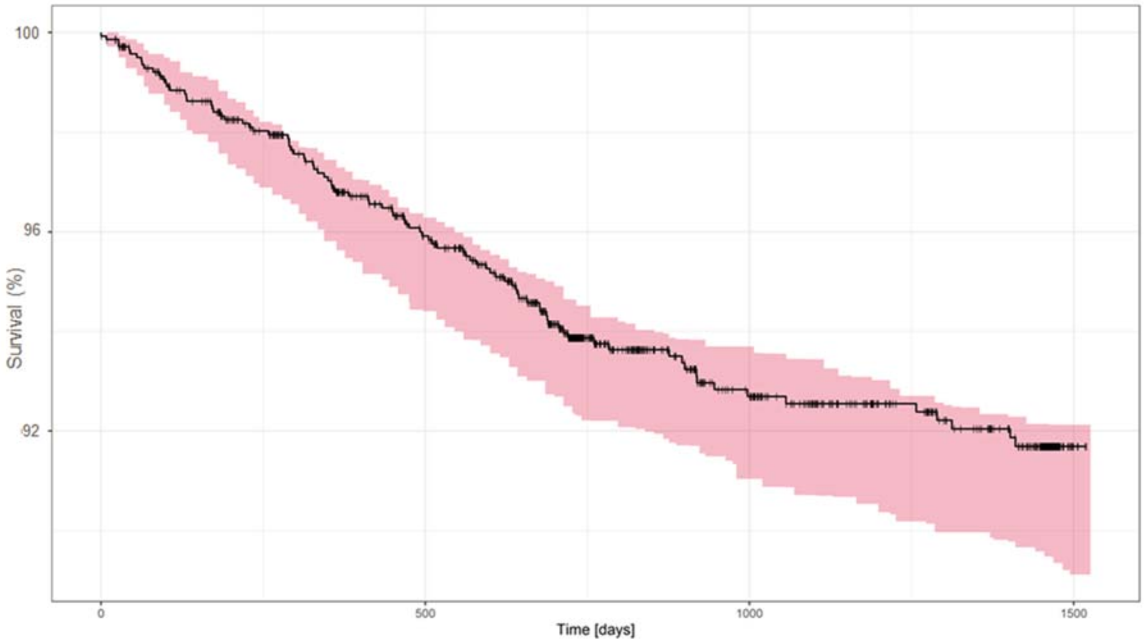

Survival (y-axis) indicates the proportion of patients who have not had an event; at time zero the survival is 100% (i.e., no patient has experienced an AUR/S). The solid line describes the observed survival over time for patients in studies ARIA3001 and ARIA3002, who switched to open label dutasteride after 24 months on placebo. Shaded areas show the model-predicted 95% confidence intervals of the survival. The impact of the disease-modifying properties of dutasteride on AUR/S is reflected by the clear inflection point at 24 months and subsequent reduction in the incidence of events.

References

1. D'Agate S, Wilson T, Adalig B, Manyak M, Palacios-Moreno JM, Chavan C, Oelke M, Roehrborn C, Della Pasqua O (2020) Model-based meta-analysis of individual IPSS trajectories in patients with benign prostatic hyperplasia with moderate or severe symptoms. Br J Clin Pharmacol. 86(8): 1585-99.

2. Roehrborn CG, Oyarzabal Perez I, Roos EPM, Calomfirescu N, Brotherton B, Wang F, Palacios JM, Vasylyev A, Manyak MJ (2015) Efficacy and safety of a fixed-dose combination of dutasteride and tamsulosin treatment (Duodart®) compared with watchful waiting with initiation of tamsulosin therapy if symptoms do not improve, both provided with lifestyle advice, in the management of treatment-naïve men with moderately symptomatic benign prostatic hyperplasia: 2-year CONDUCT study results. BJU International 116(3): 450-459.

3. Roehrborn CG, Siami P, Barkin J, Damião R, Major-Walker K, Nandy I, Morrill BB, Gagnier RP, Montorsi F (2010) The effects of combination therapy with dutasteride and tamsulosin on clinical outcomes in men with symptomatic benign prostatic hyperplasia: 4-year results from the CombAT study. Eur Urol. 57(1): 123-131.

4. Rao SR, Schoenfeld DA (2007) Survival methods. Circulation 115(1): 109-113.

5. Branson M, Whitehead J (2002) Estimating a treatment effect in survival studies in which patients switch treatment. Stats Med. 21(17): 2449-2463.

6. Morden JP, Lambert PC, Latimer N, Abrams KR, Wailoo AJ (2011) Assessing methods for dealing with treatment switching in randomised controlled trials: a simulation study. BMC Med Res Methodol. 11: 4.
